# HPV Infection Leaves a DNA Methylation Signature in Oropharyngeal Cancer Affecting Both Coding Genes and Transposable Elements

**DOI:** 10.3390/cancers13143621

**Published:** 2021-07-20

**Authors:** Diego Camuzi, Luisa Aguirre Buexm, Simone de Queiroz Chaves Lourenço, Davide Degli Esposti, Cyrille Cuenin, Monique de Souza Almeida Lopes, Francesca Manara, Fazlur Rahman Talukdar, Zdenko Herceg, Luis Felipe Ribeiro Pinto, Sheila Coelho Soares-Lima

**Affiliations:** 1 Molecular Carcinogenesis Program, Brazilian National Cancer Institute, Rio de Janeiro CEP 20231-050, Brazil; camuzi.diego@gmail.com (D.C.); labuexm@id.uff.br (L.A.B.); monique.lopes@inca.gov.br (M.d.S.A.L.); lfrpinto@inca.gov.br (L.F.R.P.); 2 Department of Pathology, Dental School, Fluminense Federal University, Rua Mario Santos Braga, 30, Centro, Niterói CEP 24040-110, Brazil; silourenco@id.uff.br; 3 Epigenetics Group, International Agency for Research on Cancer, 150 Cours Albert Thomas, CEDEX 08, 69372 Lyon, France; davide.degli-esposti@inrae.fr (D.D.E.); cuenin@iarc.fr (C.C.); manaraf@students.iarc.fr (F.M.); TalukdarF@fellows.iarc.fr (F.R.T.); HercegZ@iarc.fr (Z.H.)

**Keywords:** oropharyngeal squamous cell carcinoma, HPV, DNA methylation, transposable elements, gene expression, overall survival

## Abstract

**Simple Summary:**

The HPV oncoproteins E6 and E7 can modulate the expression and activity of the maintenance DNA methyltransferase 1, suggesting that HPV carcinogenic mechanisms may include aberrant DNA methylation. Some studies previously proposed both gene-associated DNA methylation signatures and a global hypermethylation profile in HPV-positive head and neck cancer, but the validation of such signatures and a more detailed analysis of the methylation profile of transposable elements (TEs) in oropharyngeal squamous cell carcinoma (OPSCC) are still missing. TEs account for approximately 50% of the human genome and their hypomethylation and reactivation have been consistently reported in cancer, usually being associated with worse prognosis. Based on this, this study aimed at validating a previously established 5-CpG methylation signature in FFPE OPSCC from a middle-income population, in which the frequency of HPV infection is only 6.1%, and dissecting the methylation profile of TEs, focusing on their impact on gene expression and overall survival.

**Abstract:**

HPV oncoproteins can modulate DNMT1 expression and activity, and previous studies have reported both gene-specific and global DNA methylation alterations according to HPV status in head and neck cancer. However, validation of these findings and a more detailed analysis of the transposable elements (TEs) are still missing. Here we performed pyrosequencing to evaluate a 5-CpG methylation signature and Line1 methylation in an oropharyngeal squamous cell carcinoma (OPSCC) cohort. We further evaluated the methylation levels of the TEs, their correlation with gene expression and their impact on overall survival (OS) using the TCGA cohort. In our dataset, the 5-CpG signature distinguished HPV-positive and HPV-negative OPSCC with 66.67% sensitivity and 84.33% specificity. Line1 methylation levels were higher in HPV-positive cases. In the TCGA cohort, Line1, Alu and long terminal repeats (LTRs) showed hypermethylation in a frequency of 60.5%, 58.9% and 92.3%, respectively. *ZNF541* and *CCNL1* higher expression was observed in HPV-positive OPSCC, correlated with lower methylation levels of promoter-associated Alu and LTR, respectively, and independently associated with better OS. Based on our findings, we may conclude that a 5-CpG methylation signature can discriminate OPSCC according to HPV status with high accuracy and TEs are differentially methylated and may regulate gene expression in HPV-positive OPSCC.

## 1. Introduction

Head and neck squamous cell carcinomas (HNSCC) comprise aggressive malignant neoplasms often late diagnosed with already high local invasion and metastatic dissemination [1,2]. However, given the awareness that Human Papillomavirus (HPV) infection is an important etiological factor correlating with a better therapeutic response and prognosis compared to the HPV-negative counterpart (mostly related to tobacco and alcohol), an in-depth investigation of the most relevant infection-related molecular signatures may provide new opportunities for early diagnosis and targeted therapeutic interventions [3]. HPV-positive HNSCC already represents an epidemic in high-income countries, while in low- and middle-income countries the numbers are expected to increase [4,5,6,7].

HPVs are known double-strand DNA tumor viruses, with a tropism for the basal layer of squamous epithelia, in which the completion of the viral life cycle is highly coordinated with the host keratinocyte differentiation program [8]. Among the more than 200 existing HPVs, high-risk genotype (such as HPV16 and HPV18) persistent infections progress into cancer, and in the majority of HPV-driven carcinomas, type 16 is involved [9]. Most of the HPV16-induced head and neck cancers affect the palatine tonsils, that often metastasize to nearby lymph nodes [10,11]. Crucial in the pathogenesis of HPV-driven carcinomas is the expression of early genes-encoded E6 and E7 oncoproteins which, by targeting host cell tumor suppressors p53 and pRB, provide the infected keratinocyte with a growth advantage, with enhanced proliferation rate, impaired apoptosis and progressive immune evasion [12,13]. In addition, HPV E6 and E7 can also disrupt cancer-related gene expression patterns by altering host cell transcriptional programs epigenetically, e.g., by modulating DNA methyltransferase activity [14].

DNA methylation is an epigenetic mechanism of gene expression control, and its dysregulation has been largely reported in cancer [15,16,17]. DNA methylation reactions are catalyzed by DNMTs, which in humans include four different enzymes [18]. DNMT3L lacks catalytic activity, while DNMT3A and 3B are classified as de novo DNMTs, by their capacity of methylating previously unmethylated CpG sites [18]. DNMT1, contrarily, is responsible for the maintenance of DNA methylation marks in the newly synthesized strand during DNA replication [18]. Both E6 and E7 oncoproteins affect the expression of DNMT1 (either through complex formation with E7 or as a consequence of E6-induced p53 suppression), but also of DNMT3A and DNMT3B [19,20,21,22,23]. As we recently showed, DNA methylation signatures are capable of discriminating HPV-positive and HPV-negative HNSCC, and given the different prognosis, a thorough investigation of potentially useful biomarkers allowing more effective therapeutic approaches is essential [24,25].

Although some studies reported a genome-wide hypermethylation profile based on the evaluation of transposable elements (TEs) [22,26,27,28], we showed that, in an analysis directed to gene regions, hypomethylation is more common in HPV-positive tumors relative to HPV-negative counterparts (58% and 65%, when considering differentially methylated positions and regions, respectively) [24]. This led to the identification of a 5-CpG methylation signature able to discriminate tumors according to HPV status, but the accuracy of this signature to identify HPV-driven carcinogenesis in specific tissues was not tested due to limited sample sizes.

Another important aspect yet to be investigated is the extension of the genome-wide hypermethylation profile reported in HPV-positive tumors. So far, degenerate assays for assessing Line and Alu elements methylation levels have been applied, highlighting the need for a more detailed evaluation of which TEs are dysregulated. This is especially relevant, since global hypomethylation was already associated with genomic instability and relapse in HNSCC [27,28], representing a promising biomarker. Furthermore, transposable elements also include the so-far neglected in HSCNC Long Terminal Repeat (LTR) elements, whose relevance for tumor development is starting to be unveiled [29].

In the present study, we were interested in evaluating gene-specific and global DNA methylation differences in OPSCC according to HPV status. We validated the previously proposed 5-CpG methylation signature of HPV infection in oropharyngeal squamous cell carcinoma (OPSCC) and confirmed the hypermethylation of Line1 TE in HPV-positive tumors. This led us to further dissect the methylation status of TEs, showing that, although hypermethylation is in general more common, hypomethylation is also observed. Finally, the methylation levels of gene-associated TEs were correlated with mRNA expression and impact patients overall survival, independently of HPV status.

## 2. Materials and Methods

### 2.1. OPSCC Cohort

In this study, a previously characterized [7] OPSCC cohort from the Brazilian National Cancer Institute (INCA, Rio de Janeiro, Brazil) was used. Briefly, 346 patients with a confirmed diagnosis of OPSCC between 1999–2010 were included and tumor samples were collected before any treatment. Patients’ clinical and sociodemographic information was retrieved from medical records. The median follow-up of the patients was 10.75 months. Tumor specimens were formalin-fixed and paraffin-embedded (FFPE) and representative samples from each individual were evaluated by two independent pathologists. HPV status was considered positive when a strong p16, with diffuse nuclear and cytoplasmic staining in more than 70% of the tumor cells (determined by immunohistochemistry, IHC) was observed, and HPV16 E6 DNA was detected by quantitative-PCR (qPCR). Only samples positive for the two techniques were considered HPV-positive. All other combinations, p16 negative/E6 negative, p16 positive/E6 negative, and p16 negative/E6 positive, were considered HPV-negative. This study was approved by the local Ethics Committee (60480316.0.0000.5274).

### 2.2. DNA Methylation Analyses

DNA extraction was performed and quality and quantity were assessed as previously described [7]. A total of 500 ng of genomic DNA were treated with sodium bisulfite (EpiTect Bisulfite Kit, Qiagen, Hilden, Germany) according to the manufacturer’s instructions. PCR reactions were performed to amplify the regions of interest using Platinum Taq DNA Polymerase (Invitrogen). PCR conditions as well as primer sequences can be found in Supplementary Materials Table S1.

After confirming amplification by amplicon visualization in 2% agarose gels, PCR products were pyrosequenced using PyroMark Gold Q96 Reagents (Qiagen) in a PyroMark Q96 ID system (Qiagen, Hilden, Germany). Sequencing primers can be found in Table S1. After the reaction, intensity peaks were converted to numerical values, and the methylation level of each CpG site was calculated. For the 5-CpG methylation signature, the mean methylation of the 5 CpGs analyzed (marked in bold red in Table S1) was calculated for each sample. For Line1 analysis, 5 CpG sites were assessed and the methylation levels of this TE for each patient are represented as the mean of all sites.

### 2.3. TCGA Data

Data from The Cancer Genome Atlas (TCGA) patients of the TCGA-HNSC project were retrieved. Only patients with tumors in the tonsils, base of the tongue, and oropharynx were included, totalizing 79 cases. HPV status was determined according to http://firebrowse.org/ (last accessed 10 August 2020). Patient’s clinical and sociodemographic characteristics and *CCNL1* amplification status were downloaded from cBioPortal (last accessed 7 April 2021). The median follow-up of the patients was 19.78 months in this cohort.

Expression data generated by RNA-sequencing, and methylation data generated by microarray (Infinium Human Methylation 450 K BeadChip) were obtained with the TCGAbiolinks package (v2.16.1). Normalized expression data was downloaded as fragments per kilobase million (FPKM). The raw methylation data (.idats files) were processed with the ChAMP package (v2.18.2), and the following filters were applied to remove bad quality probes: low quality probes (detection *p*-value > 0.05), cross-reactive and polymorphic probes [30]. Then, color adjustments (Lumi [31]) and normalization (BMIQ method) were applied.

### 2.4. Methylation Prediction of Repetitive Elements and Methylation Difference Analysis

The prediction analysis of the methylation levels of the transposable elements was performed with the REMP package (Repetitive Element Methylation Prediction, v1.12.0) [32] using the methylation beta values. The database for annotating repetitive elements (Line1, Alu and LTRs) was created with the initREMP function, with the UCSC data source and hg19 as the reference genome. Methylation values were preprocessed using the grooMethy function, default settings. The prediction of the elements was made by Random Forest method, with a window of 1000 bp and seed of 777. After prediction, we applied a quality filter on the elements (rempTrim function) of 1.7 and elements with a missing rate in at least 20% of the samples were removed. The methylation levels of the elements were then calculated considering elements with at least two predicted CpG methylation values (rempAggregate function).

The differential methylation analysis of the TEs between HPV-positive and HPV-negative tumors was carried out for each type of element independently with the limma package (v3.44.3) using the methylation M-value of the elements. The adjustment for multiple tests was made by the Benjamini–Hochberg FDR (BH) method.

### 2.5. Statistical Analyses

Survival analyses were performed with survival (v3.2-3) and plotted with survminer (v0.4.7) packages. For survival analyses based on gene expression, the upregulated and downregulated expression groups were determined using the median expression value for each gene in all samples. Samples with expression > median were considered upregulated and samples with expression ≤median were downregulated. Following the same approach, for the survival analysis based on Line1 methylation, hypomethylated (≤median methylation levels in all samples) and hypermethylated (> median methylation levels in all samples) cases were defined. For the estimative of univariate survival, the Kaplan−Meier survival curve was used, and statistical significance was calculated by the log-rank test. Multivariate Cox regression was applied to adjust survival for HPV status. Statistical significance was defined when *p* < 0.05.

Statistical analyses were run in an R environment or in GraphPad Prism 5 software (GraphPad Software, San Diego, CA, USA). The correlation between the methylation levels of the TEs mapped to promoter regions and the expression of the associated genes was performed by Spearman’s test. Comparisons between groups were performed using the Mann−Whitney test. When necessary, *p*-value adjustment was performed by the BH method. All these analyses were made with base (v4.0.5) and stats (v4.0.5) R packages.

## 3. Results

### 3.1. HPV Infection Is Associated with a Specific DNA Methylation Signature and Global Methylation in OPSCC

**Figure 1:**
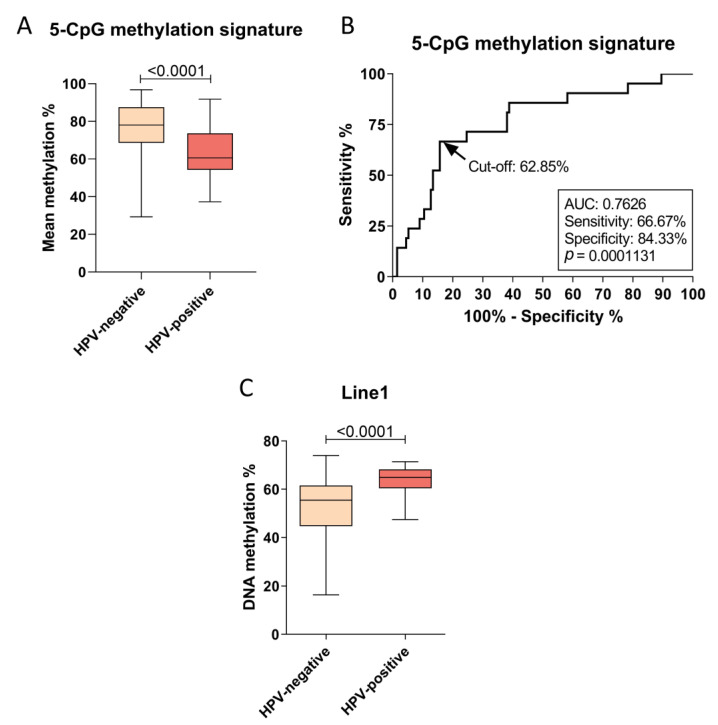
DNA methylation signatures of HPV-positive OPSCC. (**A**) Boxplot showing the mean methylation of 5 CpG sites in *B3GALT6*-*SDF4*, *SYCP2*-*FAM217B*, and *HLTF*-*HLTF-AS1* loci assessed by pyrosequencing in HPV-positive (*n* = 21) and HPV-negative (*n* = 134) OPSCC. (**B**) Receiver operating characteristic (ROC) curve showing the best DNA methylation cut-off of the 5-CpG signature to distinguish HPV-positive and HPV-negative OPSCC. (**C**) Boxplot showing the methylation levels of Line1 transposable element assessed by pyrosequencing in HPV-positive (*n* = 21) and HPV-negative (*n* = 325) OPSCC.

In this study, we used a previously characterized cohort of OPSCC patients that showed a low frequency of HPV-positive cases (6.1%), assessed by both p16 IHC and HPV DNA detection by qPCR [7]. Our data show that the mean methylation levels of 5 CpG sites located at *B3GALT6*-*SDF4*, *SYCP2*-*FAM217B*, and *HLTF*-*HLTF-AS1* loci [24] are lower in HPV-positive relative to HPV-negative tumors (median of 60.62% vs. 78.07%, *p* < 0.001, Figure 1A). The DNA methylation levels of each CpG site composing the signature are shown in Figure S1. After applying the previously suggested DNA methylation cut-off (<75%) [24], this 5-CpG signature was able to discriminate OPSCC according to HPV status with 80.95% sensitivity and 61.2% specificity. Since the methylation cut-off was previously determined in HNSCC in general, and the sample source differed from the present study (fresh-frozen and FFPE, respectively), we applied a receiver operating characteristic (ROC) curve analysis to test new cut-offs in our experimental settings (Figure 1B). This analysis showed that with a DNA methylation cut-off of 62.85%, the methylation signature was able to discriminate the groups with 66.67% sensitivity and 84.33% specificity (*p* = 0.0001, Figure 1B).

Apart from gene-specific signatures, HPV infection was also previously associated with global methylation dysregulation [22,26,27,28]. To test this hypothesis, we assessed Line1 methylation in our sample set. Figure 1C shows that Line1 methylation levels are higher in HPV-positive relative to HPV-negative OPSCC (median of 64.89% vs. 55.47%, *p* < 0.0001, Figure 1C). However, overall survival (OS) of the cases did not differ according to Line1 methylation levels (Figure S2).

Although Line1 elements have been assessed as a proxy of global methylation, other transposable elements also represent an important percentage of the human genome, including Alu and LTRs. These elements can be found both in intergenic and intragenic regions, and their methylation levels may impact prognosis. Based on this, we next evaluated the methylation levels of all these elements in OPSCC.

### 3.2. Transposable Elements Are Differentially Methylated According to HPV Status in OPSCC

**Figure 2:**
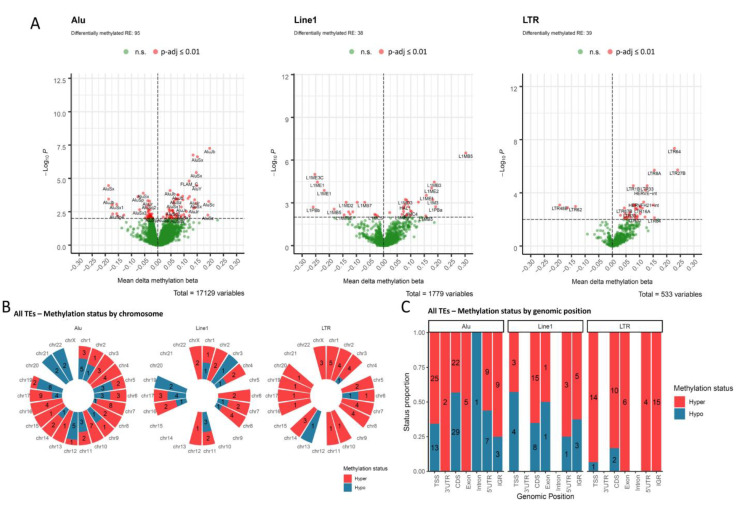
Transposable elements are differentially methylated in OPSCC according to HPV status. (**A**) Volcano plots showing the Line1, Alu and LTR elements differentially methylated in HPV-positive relative to HPV-negative OPSCC. The X-axis shows the mean beta value differences (mean methylation delta) between groups and the Y-axis shows the -Log Benjamini-Hochberg (BH) adjusted *p*-values. Red dots represent elements with BH adjusted *p*-value ≤ 0.01. (**B**) Chromosomal distribution and (**C**) genomic region distribution of hypermethylated (red) and hypomethylated (blue) Line1, Alu and LTR elements in HPV-positive relative to HPV-negative OPSCC. The same TE may encompass more than one genomic region.

We extracted DNA methylation information of TEs from TCGA OPSCC cohort and compared each individual element according to HPV status. Our data show that a total of 172 TEs are differentially methylated in HPV-positive relative to HPV-negative tumors (BH adjusted *p*-value ≤ 0.01), with hypermethylation being more commonly observed in HPV-positive cases. The frequency, however, varied according to TE type (Figure 2A), for Alu and Line1 a similar proportion of hypermethylated elements was observed (58.9% and 60.5%, respectively), while for LTRs this proportion was higher (92.3%).

Differentially methylated TEs were not individually enriched in any specific chromosome (Figure 2B). The stratification by genomic region reproduced the overall profile, with most of the elements showing hypermethylation in HPV-positive OPSCC in any given region (Figure 2C). The exceptions were Alu elements located in coding sequences and Line1 elements located in promoters (up to 2000 bp from transcription start sites), with 56.9% and 57.1% of the differentially methylated elements showing hypomethylation, respectively (Figure 2C). Alu, Line1 and LTR mapped to intergenic regions were mostly hypermethylated in HPV-positive relative to HPV-negative tumors (75%, 62.5% and 100%, respectively).

### 3.3. The Methylation Levels of Transposable Elements Mapped to Promoter Regions Are Correlated with Expression and Prognosis

**Figure 3:**
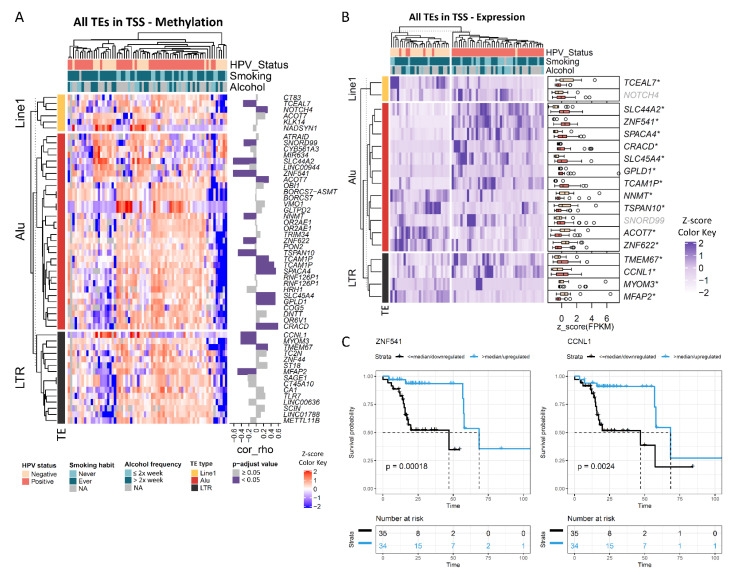
Differentially methylated transposable elements located at promoter regions show a correlation with gene expression and impact on prognosis. (**A**) Heatmap showing the unsupervised clustering of OPSCC TCGA samples according to the DNA methylation profile of transposable elements located up to 2000 bp of transcription start sites (promoter regions). Each line represents a transposable element and each column represents a sample. On the right, bar plots show the correlation rho (Spearman test) between the methylation levels of the element and the expression of the associated gene. Dark and light purple bars represent significant (BH adjusted *p* < 0.05) and nonsignificant correlations, respectively. (**B**) Heatmap showing the clustering of OPSCC TCGA samples according to the expression levels of genes correlated with the methylation levels of transposable elements in their promoter regions. Each line represents a transposable element and each column represents a sample. On the right, boxplots showing the expression of each gene in HPV-positive (dark pink) and HPV-negative (light pink) OPSCC. * Genes differentially expressed according to HPV status (BH adjusted *p* < 0.05). (**C**) Kaplan−Meier curves showing the overall survival of OPSCC patients from TCGA cohort according to the expression of *ZNF541* and *CCNL1*. High and low expression were defined according to the median expression of each gene in all samples.

Figure 3A shows the methylation profile of TEs mapped to promoter regions. Since this analysis indicated a heterogeneous profile among HPV-positive cases, we performed unsupervised clustering of these tumors (Figure S3). Although two DNA methylation clusters were identified, they were not associated with age, tobacco smoking, tumor stage or location.

We next evaluated whether DNA methylation alterations in TEs mapped to promoters were associated with gene expression. For Alu elements, the methylation levels of 13 out of 32 elements (40.6%) showed a significant correlation with the mRNA expression of the associated gene (Figure 3A). The correlation was inverse for *SNORD99*, *SLC44A2*, *ZNF541*, *NNMT*, *ZNF622* and *TSPAN10*, and positive for *ACOT7*, *TCAMP1*, *SPACA4*, *SLC45A4*, *GPLD1* and *CRACD*. From a total of six differentially methylated Line1 elements mapped to promoter regions, two (33.3%) showed a significant correlation with gene expression, being inverse for *TCEAL7* and positive for *NOTCH4* (Figure 3A). The methylation levels of LTR elements were also significantly correlated with expression in 26.7% of the genes (4 out of 15) (Figure 3A). The correlation was inverse for *CCNL1*, *MYOM3* and *MFAP2*, and positive for *TMEM67*.

Figure 3B shows the expression of those genes that showed a significant correlation with TEs methylation levels, according to HPV status in TCGA OPSCC cohort. Downregulated and hypermethylated genes in HPV-positive versus HPV-negative OPSCC included *TCEAL7*, *NNMT*, *TSPAN10*, *ZNF622*, *MYOM3* and *MFAP2*. *ACOT7* was also downregulated, but its promoter was hypomethylated in HPV-positive cases. Among the upregulated genes, *SLC44A2*, *ZNF541* and *CCNL1* were also hypomethylated, while *SPACA4*, *CRACD*, *SLC45A4*, *GPLD1*, *TCAMP1* and *TMEM67* were hypermethylated (Figure 3B). The correlation plots between DNA methylation and mRNA expression for *CCNL1* and *ZNF541* are shown in Figure S4.

Finally, the association between the expression of these genes and overall survival was assessed (Table S2). After adjustment for HPV status, patients with high *ZNF541* or *CCNL1* expression showed a median OS of 68.4 months vs. 47 months in the group with low expression (HR = 0.17, 95% CI 0.03–0.91 and HR = 0.34, 95% CI 0.13–0.93, respectively) (Figure 3C, Table S2). Since *CCNL1* amplification was previously associated with worse outcomes in head and neck cancer [33], we evaluated whether this could explain the differences we found in OPSCC. However, we did not observe a different frequency of *CCNL1* amplification according to HPV status (21% in HPV-negative and 18% in HPV-positive) nor a significant impact of *CCNL1* amplification on OS (Figure S5).

## 4. Discussion

HPV oncoproteins were shown to modulate DNMT1 levels [19,20,34] and some studies have been developed to identify HPV-associated alterations of DNA methylation in cervical cancer [35,36,37]. This led to the design of gene panels whose methylation levels can discriminate tumors from non-tumor tissues as well as predict preneoplastic lesions more likely to progress, with high accuracy [35,38,39,40]. However, in head and neck cancer, the association between HPV infection and DNA methylation aberration has been less explored, both genome-wide and in target genes. Although DNA methylation signatures of infection and a global hypermethylation phenotype have been proposed in HNSCC [22,24,25,26,28], the validation of these signatures, and a more detailed characterization of the affected regions are still missing. Here we show that a previously proposed 5-CpG methylation signature can discriminate HPV-positive and HPV-negative OPSCC from a population with a low frequency of HPV positivity (which usually hampers biomarker sensitivity) and using a different sample source. We also show that transposable elements are mostly hypermethylated in HPV-positive relative to HPV-negative OPSCC. However, hypomethylated TEs mapped to promoter regions lead to the upregulation of associated genes, with an impact on OS, independent of HPV infection.

P16 IHC is the most widely used biomarker in the context of HPV-associated carcinogenesis. In a recent meta-analysis, its sensitivity and specificity to detect cervical intraepithelial neoplasia of grade 2 (CIN2^+^), 3 or worse (CIN3^+^) ranged from 82–86% and 49–71%, respectively [41]. When combined with Ki67 IHC, p16 showed a sensitivity of 75.2% to detected CIN2^+^, compared to 61.0% achieved by cytology alone [42]. In HNSCC, p16 IHC sensitivity to detect HPV-positive cases is high (92%, 95% CI 82–97%), while its specificity seems to be moderate (72%, 95% CI 45–89%) [43]. In OPSCC, p16 was able to identify HPV-driven transformation with a sensitivity of 94% (95% CI 91–97%) and a specificity of 83% (95% CI 78–88%) [44]. Although the sensitivity of the 5-CpG methylation signature tested here was lower when compared to p16 IHC, we were able to reproduce the previous results obtained in high-income populations [24] in a middle-income population, in which the frequency of HPV-positive cases is more modest [7]. This was also the first time this signature established in all HNSCC subsites was tested in OPSCC and FFPE samples. This is relevant because an optimal biomarker should perform well independently of sample source or experimental assay. By assessing the methylation levels of a diagnostic signature in paired exfoliated cells and FFPE biopsies from women referred to colposcopy, Reuter and colleagues showed a correlation ranging from 0.379–0.550 between tests. FFPE cut-off adjustments were also necessary to improve the signature accuracy [45]. Therefore, the previously proposed HPV 5-CpG methylation signature is reproducible in OPSCC from a population with a low frequency of HPV infection and in FFPE, a more easily available source of tumor samples.

DNA methylation signatures have emerged as promising biomarkers in oncology capable of measuring risk factor exposure [35,46,47], discriminating different types of primary tumors [48,49], predicting the progression of preneoplastic lesions [50,51], predicting response to treatment and prognosis [35,52,53,54], and even detecting malignant tumors years before conventional diagnosis [55]. Although different DNA methylation signatures are usually assessed for different tissues and outcomes, a common feature of cancers is global hypomethylation [17,56,57], in general associated with poor OS [58,59,60,61]. Therefore, we also assessed this profile in our samples, and showed higher Line1 methylation levels in HPV-positive relative to HPV-negative OPSCC, corroborating previous data [22,26,27,28]. Although we did not observe an association between Line1 methylation levels and OS, it is important to mention that for the same population studied here, HPV was not associated with prognosis, likely due to the high frequency of smokers [7].

The association between global DNA hypomethylation and poor prognosis is usually linked to genomic instability [28,62,63,64]. Almost 50% of the human genome is composed of transposable elements, which are silenced by hypermethylation in normal cells [65,66]. During transformation, TEs lose methylation, and are therefore reactivated, leading to their mobilization [65,67]. Based on this, we evaluated whether the global hypermethylation in HPV-positive relative to HPV-negative OPSCC assessed by Line1 methylation was common to all TEs. In general, hypermethylation in HPV-positive tumors was observed for Line1, Alu and LTR. The frequency, however, differed among these classes of TEs. LTRs, which include the endogenous retrovirus (ERVs), showed the highest hypermethylation proportion. ERV activation by demethylating agents was shown to induce viral mimicry and, therefore, activate the immune response [68,69,70]. Since these elements were shown here to be widely hypermethylated in HPV-positive OPSCC, although speculative, this might be a mechanism by which HPV guarantees immune escape.

Although the majority of TEs are mapped to intergenic regions, their participation in regulating gene expression has been proposed [71]. Different mechanisms such as control of transcription by DNA methylation, chromatin remodeling, recruitment of transcription factors, generation of new isoforms, and even the regulation of translation put TEs as relevant regulators of cell behavior [66,71]. As an example, Line1 methylation was already associated with the regulation of the expression of genes with specialized functions, while SINEs methylation regulated the expression of genes involved in housekeeping activities in embryonic stem cells [72]. By assessing whether the methylation of these elements was correlated with gene expression, we showed a significant correlation for about one third of the genes analyzed. This was further corroborated by the differential expression of these genes in HPV-positive relative to HPV-negative OPSCC. These results suggest that the differential methylation of TEs according to HPV status may have an impact on tumor cells that goes beyond genetic instability.

Among the hypermethylated and downregulated genes, *TCEAL7* is a putative tumor suppressor gene downregulated by DNA methylation in ovarian cancer [73]. It associates with promoters containing MYC and NF-κB binding sites, such as *cyclin D1* promoter, repressing transcription. Therefore, *TCEAL7* downregulation was proposed as an alternative mechanism for the activation of MYC and NF-κB target genes [73,74]. NNMT upregulation was already reported in oral squamous cell carcinoma [75], but its downregulation was associated with increased sensitivity to 5-fluorouracil in esophageal squamous cell carcinoma cells [76]. In contrast, *MFAP2* upregulation was reported in head and neck cancer [77] and associated with poor prognosis in gastric and hepatocellular carcinomas [78,79]. Therefore, the downregulation of these genes in HPV-positive relative to HPV-negative OPSCC may contribute to the better prognosis and response to therapy observed in the first group of patients. Although an association between *ZNF622* and cancer has not been reported yet, its role as an antiviral protein upon adenovirus infection was proposed [80]. Based on these evidences, the dysregulation of gene expression via the differential methylation of TEs in HPV-positive OPSCC may contribute to successful HPV infection and carcinogenicity, but future studies are necessary to corroborate this hypothesis.

The expression of *ZNF541* and *CCNL1*, both upregulated and hypomethylated in HPV-positive cases, were associated with OS independently of HPV status. *ZNF541* encodes a zinc finger protein supposed to be a component of chromatin remodeling complexes and previously suggested to play a role in the differential expression of genes according to HPV status in cervical cancer tissues and cell lines [81]. *CCNL1* encodes Cyclin L1 and its role as an oncogene in head and neck cancer by promoting cell cycle entry was previously proposed [82]. The so far recognized mechanism behind *CCNL1* overexpression was gene amplification, which was associated with lymph node metastasis in HNSCC [33]. However, the expression differences we observed in OPSCC according to HPV status do not seem to be associated with *CCNL1* amplification, but with promoter-associated LTR methylation, suggesting a new mechanism behind its transcriptional regulation. Recently, *CCNL1* promoter differential methylation was associated with platinum resistance in ovarian cancer, but its impact on gene expression was not evaluated [83]. Therefore, our results bring a new connection between HPV infection, TEs methylation, regulation of gene expression and prognosis.

Finally, we also observed DNA methylation differences of promoter-associated TEs within HPV-positive cases, but no significant associations with age, tobacco smoking, tumor stage or location were found. However, the sample size included was limited and other biological mechanisms not evaluated here might contribute to these differences. For example, HPV integration was already associated with a specific DNA methylation signature in HNSCC [84]. Future studies are necessary to explore whether HPV integration also affects the methylation profile of transposable elements.

## 5. Conclusions

Here we validated a previously proposed 5-CpG methylation signature of HPV infection. This signature was originally established in head and neck cancer from high-income populations using snap frozen samples, and we were able to replicate the original findings in OPSCC from a middle-income population (which shows a frequency of HPV positivity of only 6.1%) using FFPE samples. We also showed that global methylation, estimated by Line1 assessment, is higher in HPV-positive relative to HPV-negative OPSCC. This led us to investigate the methylation profile of transposable elements in depth, showing that not only Line1, but also Alu and LTRs are hypermethylated in HPV-positive cases. However, the hypermethylation frequency varied according to TE class and genomic region. Finally, we evaluated the correlation between the methylation levels of TEs mapped to promoter regions and the expression of the associated genes, showing significant correlations for approximately one third of the genes. Among those genes differentially expressed according to HPV status, *ZNF541* and *CCNL1* (harboring Alu and LTR in their promoters, respectively) higher expression was significantly associated with a better overall survival, independent of HPV status.

## Data Availability

Publicly available RNA-seq and DNA methylation data from oropharyngeal cancer patients from the Cancer Genome Atlas (TCGA-HNSC project) were retrieved from cBioPortal.

## Supplementary Materials

The following are available online at https://www.mdpi.com/article/10.3390/cancers13143621/s1, Figure S1: Boxplot showing the methylation levels of the individual CpG sites that compose the 5-CpG methylation signature, according to HPV status in INCA cohort. Figure S2: Kaplan−Meier plot showing the overall survival of OPSCC patients from our cohort according to Line1 methylation. Figure S3: DNA methylation profile of TEs mapped to promoters in HPV-positive OPSCC. Figure S4: Analysis of the correlation between the DNA methylation levels of the transposable elements mapped to the promoters of *CCNL1* and *ZNF541* and the mRNA expression of the associated genes in TCGA cohort. Figure S5: Kaplan−Meier plot showing the overall survival of OPSCC patients from TCGA cohort according to the presence of *CCNL1* amplification. Table S1: Primers sequences and PCR conditions used in the present study. Table S2: Multivariate Cox regression analysis of the impact of the expression of genes harboring transposable elements in their promoters on overall survival of OPSCC patients from TCGA cohort.
