# Supplementary material for: HPV Infection Leaves a DNA Methylation Signature in Oropharyngeal Cancer Affecting Both Coding Genes and Transposable Elements
Source: Cancers (Basel). 2021 Jul 20;13(14):3621. doi: 10.3390/cancers13143621 (PMC8306428; doi:10.3390/cancers13143621)
Supplement: Supplementary file 1 [file cancers-13-03621-s001.zip › Supplementary Materials_post revision.pdf]

cancers

MDPI

Supplementary Materials:

Figure S1: Boxplot showing the methylation levels of the individual CpG sites that compose the 5-CpG methylation signature, according to HPV status in INCA cohort.

Figure S2: Kaplan Meier plot showing the overall survival of OPSCC patients from our cohort according to Line1 methylation. Hypomethylated and hypermethylated cases were defined according to the median methylation of Line1 in all samples.

Figure S3: DNA methylation profile of TEs mapped to promoters in HPV-positive OPSCC. Heatmap showing the unsupervised clustering of HPV-positive OPSCC according to the DNA methylation levels of promoter-associated transposable elements. Two clusters were defined and are indicated in the Figure.

Figure S4: Analysis of the correlation between the DNA methylation levels of the transposable elements mapped to the promoters of *CCNL1* and *ZNF541* and the mRNA expression of the associated genes in TCGA cohort.

Figure S5: Kaplan Meier plot showing the overall survival of OPSCC patients from TCGA cohort according to the presence of *CCNL1* amplification.
