# Supplementary figures and images for: HPV Infection Leaves a DNA Methylation Signature in Oropharyngeal Cancer Affecting Both Coding Genes and Transposable Elements
Source: Cancers (Basel). 2021 Jul 20;13(14):3621. doi: 10.3390/cancers13143621 (PMC8306428; doi:10.3390/cancers13143621)

# 5-CpG methylation signature

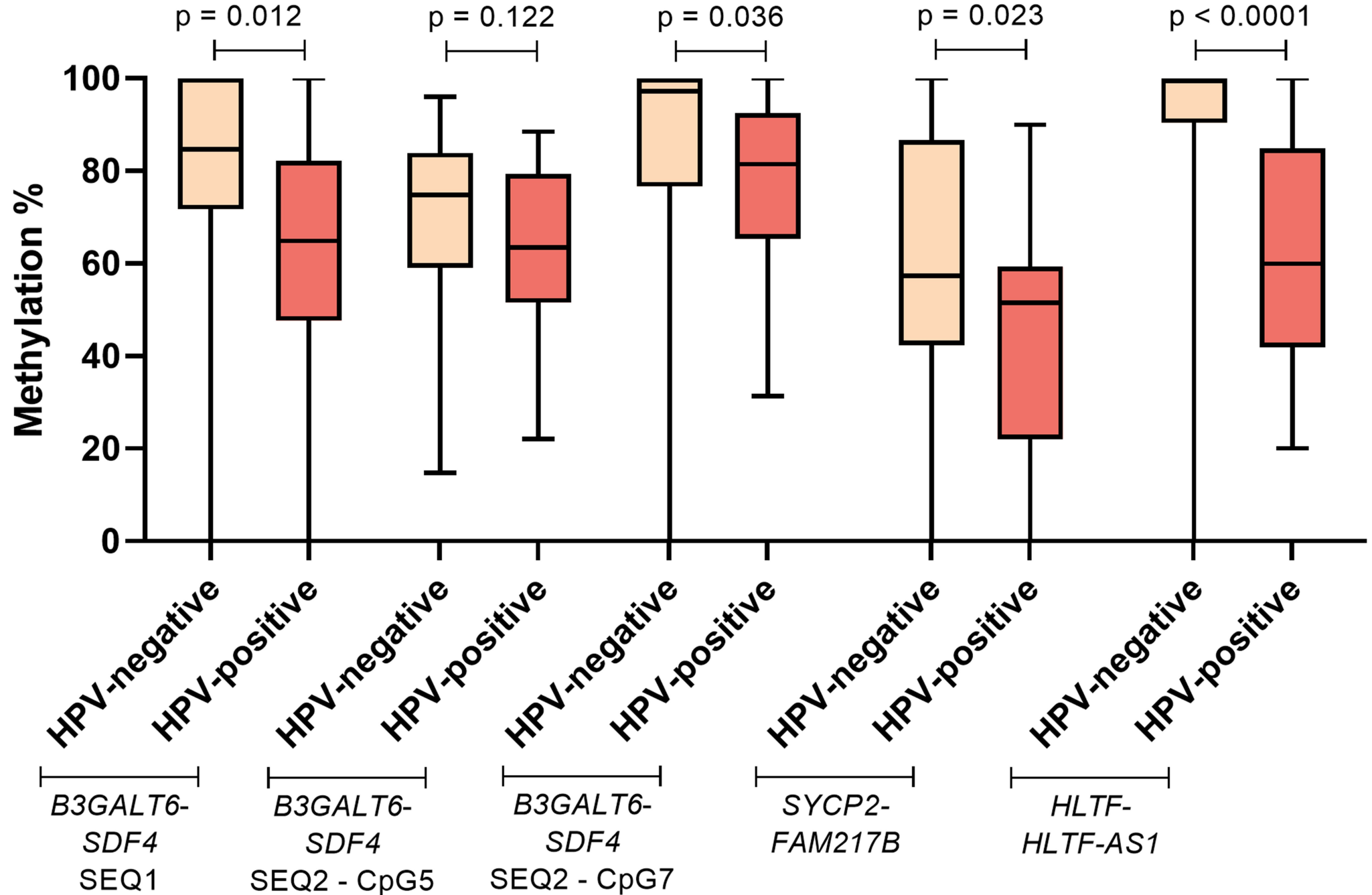

Supplement: Supplementary file 1 [file cancers-13-03621-s001.zip › Figure S1.pdf]

## OS according to LINE1 methylation (median)

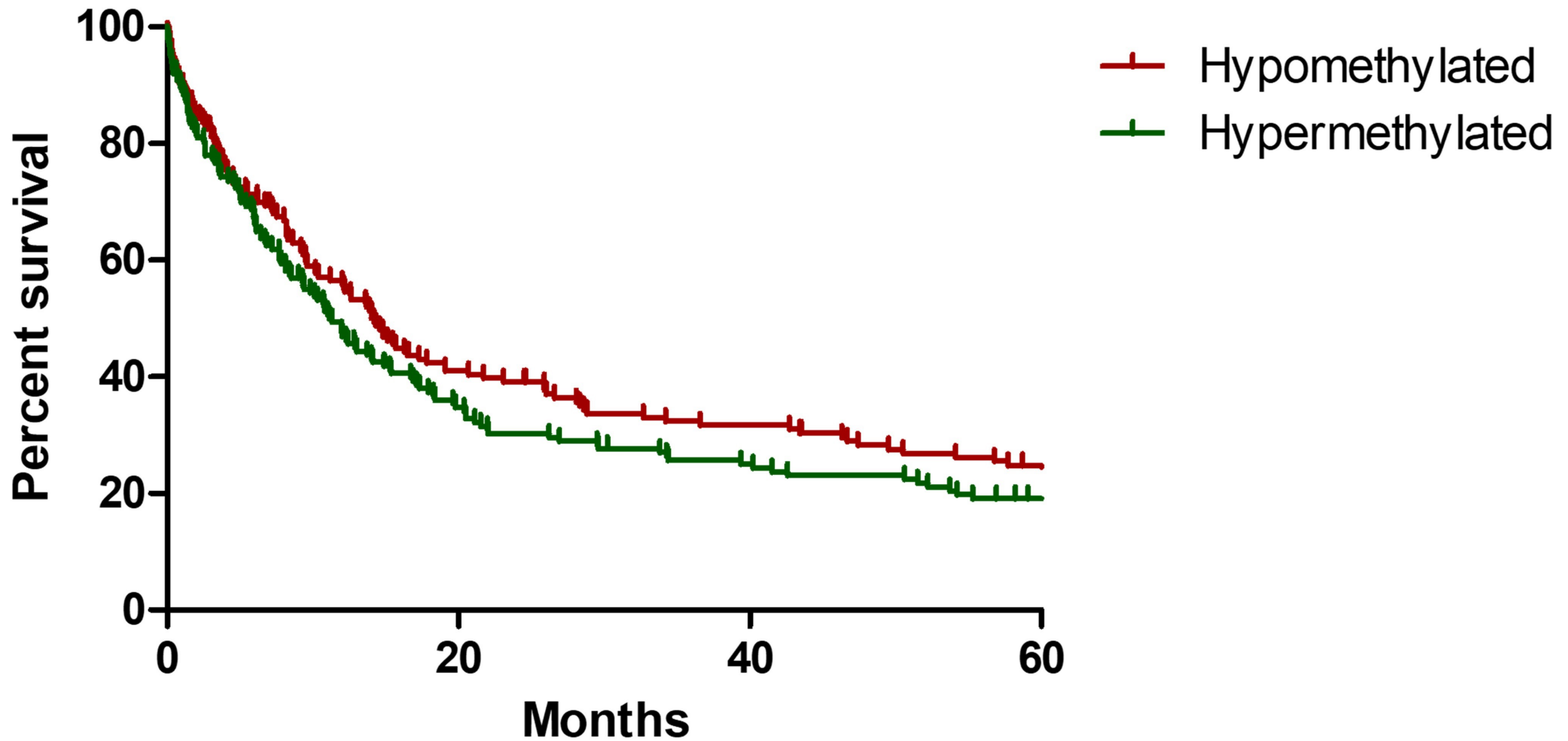

Supplement: Supplementary file 1 [file cancers-13-03621-s001.zip › Figure S2.pdf]

# All TEs in TSS - Methylation

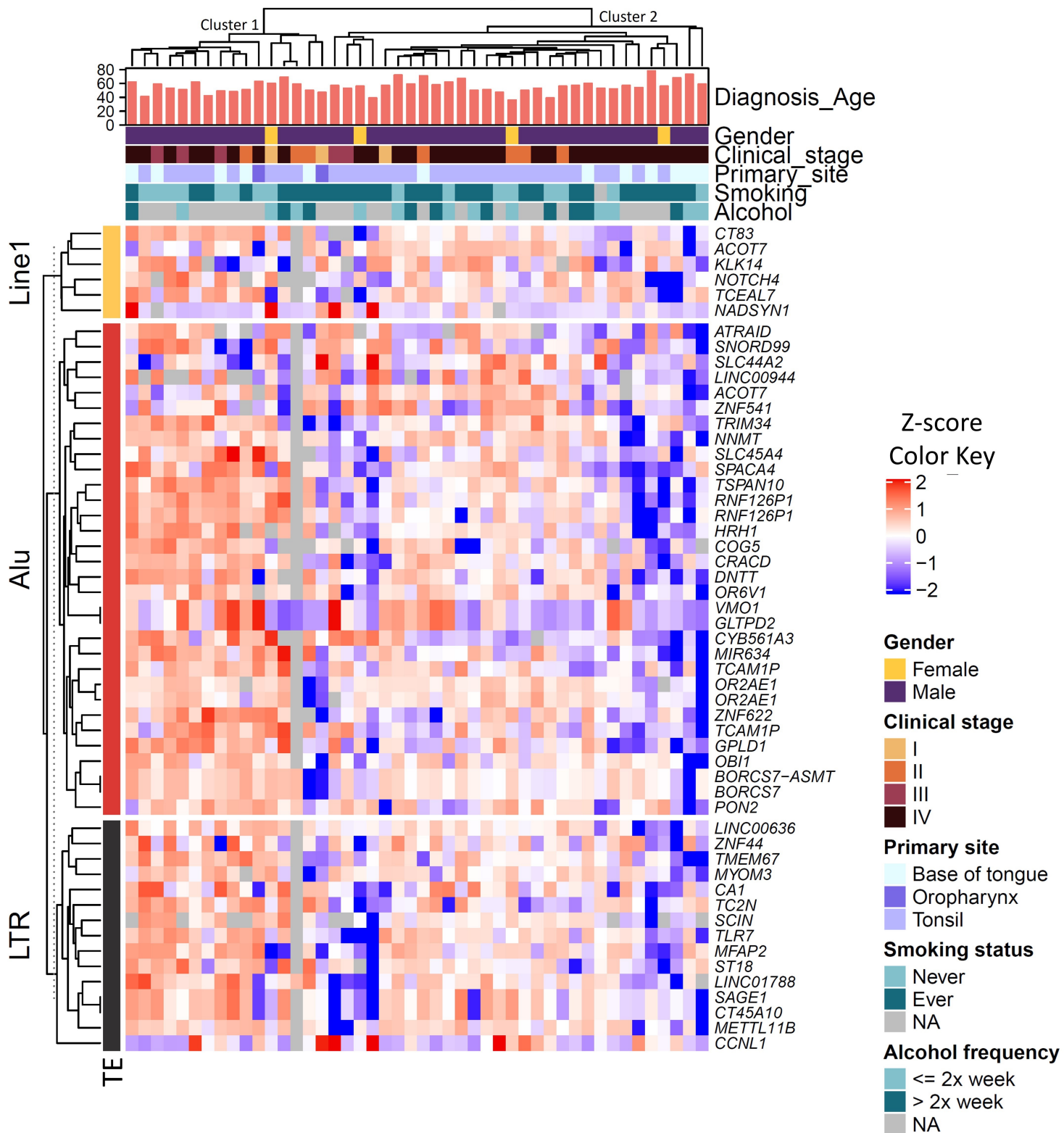

Supplement: Supplementary file 1 [file cancers-13-03621-s001.zip › Figure S3.pdf]

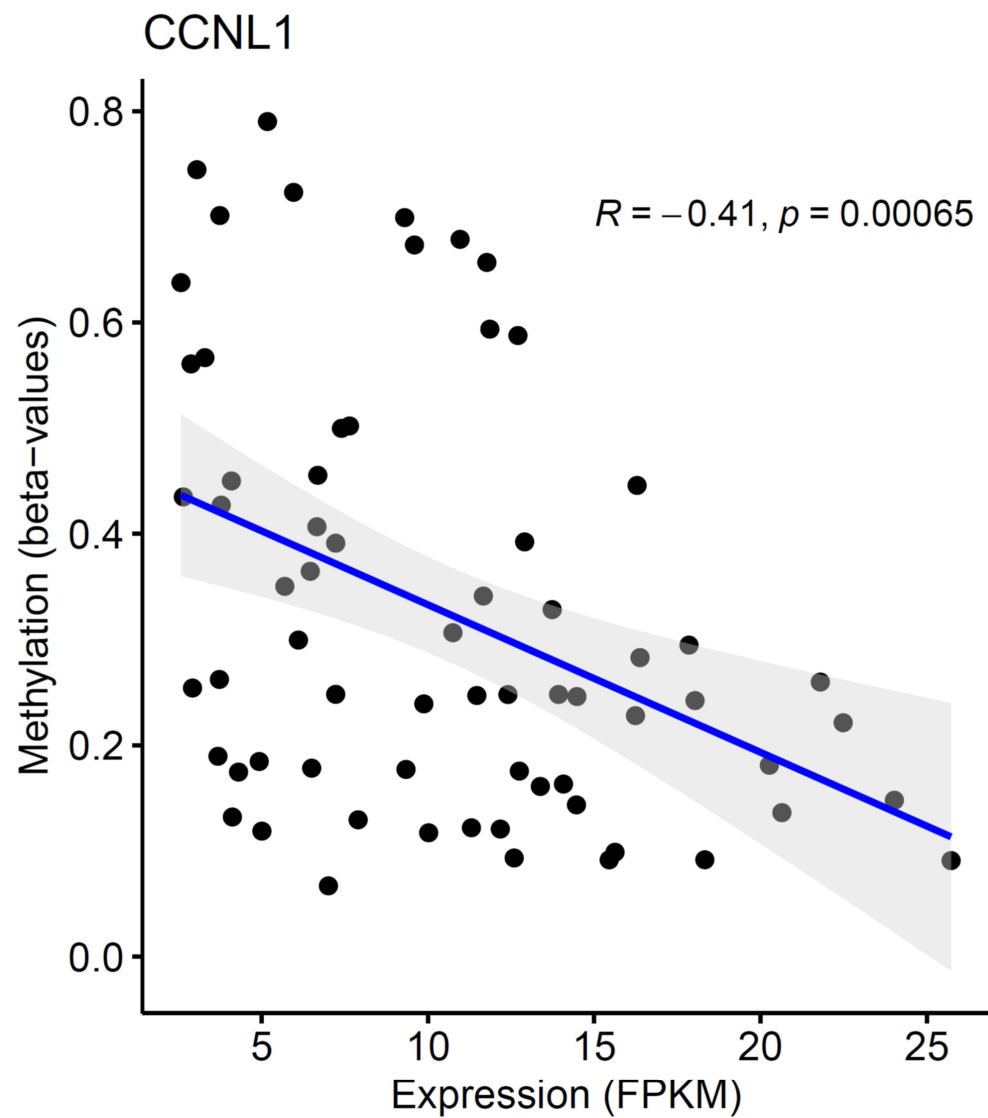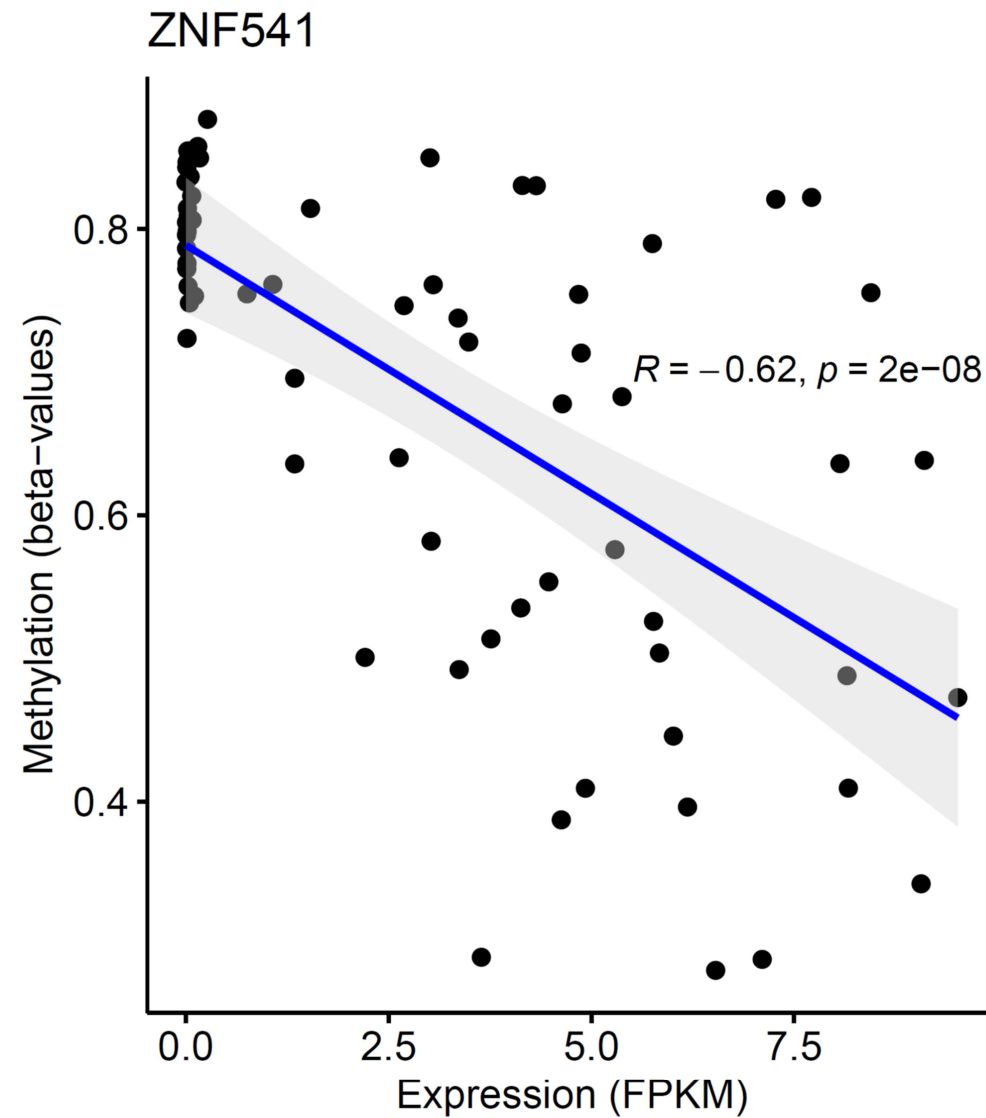

Supplement: Supplementary file 1 [file cancers-13-03621-s001.zip › Figure S4.pdf]

# CCNL1 status

Strata AMP no alterations

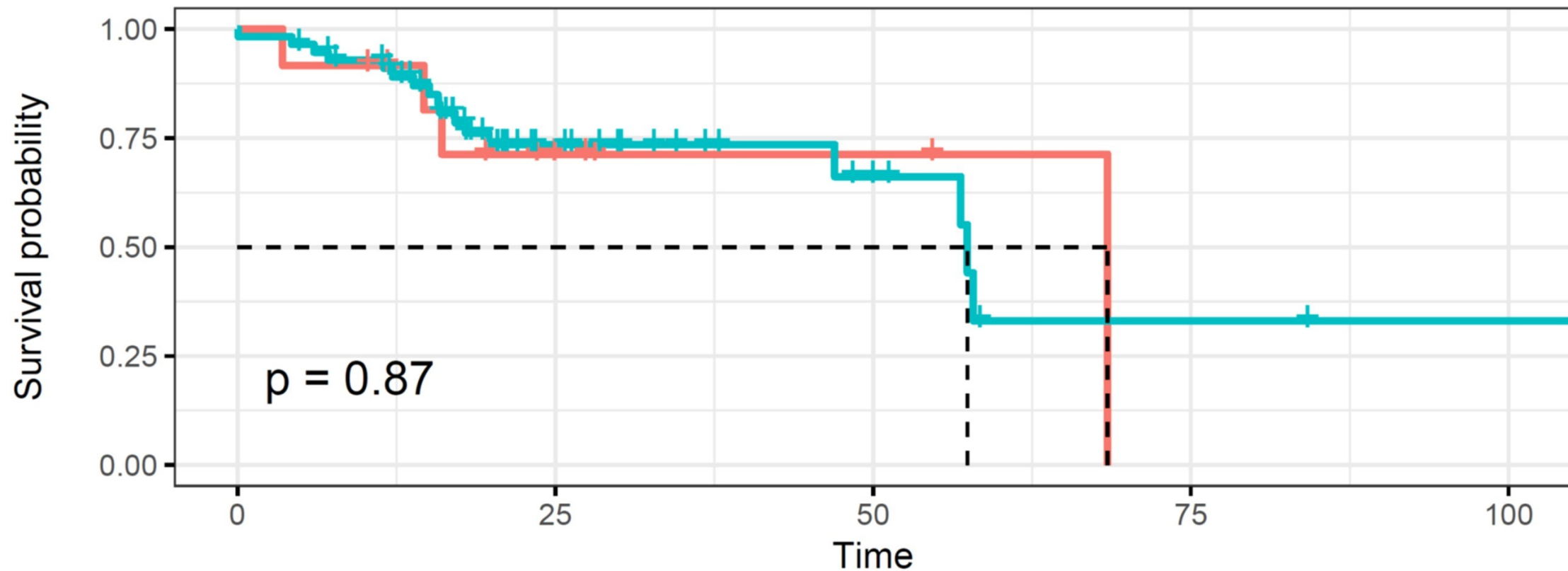

## Number at risk

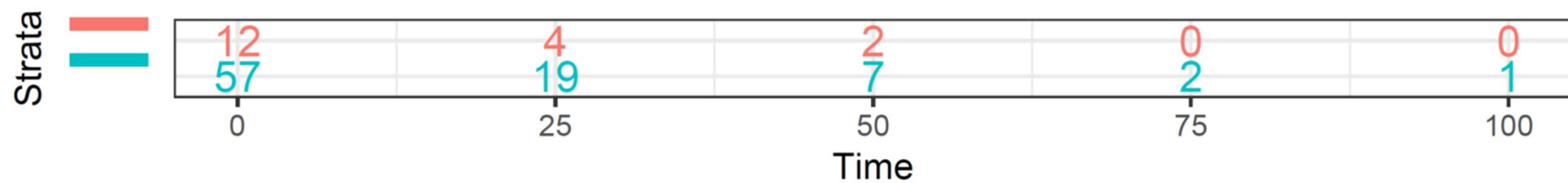

Supplement: Supplementary file 1 [file cancers-13-03621-s001.zip › Figure S5.pdf]
